# Understanding measures of racial discrimination and microaggressions among American Indian and Alaska Native college students in the Southwest United States

**DOI:** 10.1186/s12889-021-11036-9

**Published:** 2021-06-09

**Authors:** Brenna L. Greenfield, Jessica H. L. Elm, Kevin A. Hallgren

**Affiliations:** 1 Department of Family Medicine & Biobehavioral Health, University of Minnesota Medical School, Duluth Campus, 1035 University Drive, Duluth, MN 55812 USA; 2 Great Lakes Hub, Center for American Indian Health, Department of International Health, Bloomberg School of Public Health, Johns Hopkins University, Baltimore, MD USA; 3 Department of Psychiatry & Behavioral Sciences, University of Washington, Box 356560, 1959 NE Pacific Street, Seattle, Washington 98195 USA

**Keywords:** American Indian and Alaska Native, Racial discrimination, Microaggressions, Item response theory, College students

## Abstract

**Background:**

Racial discrimination, including microaggressions, contributes to health inequities, yet research on discrimination and microaggressions has focused on single measures without adequate psychometric evaluation. To address this gap, we examined the psychometric performance of three discrimination/microaggression measures among American Indian and Alaska Native (AI/AN) college students in a large Southwestern city.

**Methods:**

Students (*N* = 347; 65% female; ages 18–65) completed the revised-Everyday Discrimination Scale, Microaggressions Distress Scale, and Experiences of Discrimination measure. The psychometric performance of these measures was evaluated using item response theory and confirmatory factor analyses. Associations of these measures with age, gender, household income, substance use, and self-rated physical health were examined.

**Results:**

Discrimination and microaggression items varied from infrequently to almost universally endorsed and each measure was unidimensional and moderately correlated with the other two measures. Most items contributed information about the overall severity of discrimination and collectively provided information across a continuum from everyday microaggressions to physical assault. Greater exposure to discrimination on each measure had small but significant associations with more substance use, lower income, and poorer self-rated physical health. The Experiences of Discrimination measure included more severe forms of discrimination, while the revised-Everyday Discrimination Scale and the Microaggressions Distress Scale represented a wider range of severity.

**Conclusions:**

In clinical practice, these measures can index varying levels of discrimination for AI/ANs, particularly for those in higher educational settings. This study also informs the measurement of racial discrimination and microaggressions more broadly.

**Supplementary Information:**

The online version contains supplementary material available at 10.1186/s12889-021-11036-9.

## Background

For centuries, pervasive race-based discrimination has caused harm to Indigenous peoples of Turtle Island [11]. Colonial settlers of what would come to be known as North America brought with them attitudes of greed and supremacy, drive for acquisition of natural resources that were not their own, and desire for free labor. The result was colonization, genocide, and slavery. Settler colonialism, including continued drive for power and privilege, led to contemporary forms of oppression (e.g., systemic racism [65];). Today, interlocking systemic processes are necessary for ongoing domination of Black, Indigenous, and People of Color (BIPOC [20];) and BIPOCs experience a range of interpersonal and environmental expressions of racism from violent acts to unconscious bias and microaggressions. Naming these experiences is one step towards addressing them, and towards healing.

Racial discrimination is one manifestation of racism, described as “a behavioral manifestation of a negative attitude, judgment, or unfair treatment toward members of a group” ([40]; p. 533). Commonly, racial discrimination occurs in the form of microaggressions, described as brief, daily battles [12] or a chronic contemporary sub-type of discrimination (Lee & Turney, 2012 [34]; Hollingsworth et al., 2017 [22]).

Microaggressions can be subtle, intentional or unintentional slights and insults that communicate hostility and inferiority toward a target person or group [41, 54].

### Discrimination and health

Exposures to discrimination, including microaggressions, are disproportionately reported by BIPOCs [2, 13] and likely contribute to racial and ethnic education and health disparities (Lewis et al., 2015 [36, 52];). Exposures can range in magnitude and frequency from an uncommon traumatic incident with lasting impact to frequent interruptions and annoyances in daily life ([12]; Schmitt, 2014 [48]). The harm caused by discrimination operates through various mechanisms and occurs across contexts and key life domains (e.g., institutional racism; workplace, health care services [12]; Hollingsworth et al., 2017 [22]; O’Keefe et al., 2015 [38, 63];). For example, at the individual-level, microaggressions as psychosocial stressors can accumulate over time, with little opportunity for resolution ([12]; Lee & Turney, 2012 [34]). Anxiety [24, 39], early substance use [14], and physical health indicators, such as increased blood pressure and systemic inflammation [18] are also related to discrimination. Another example of how discrimination may impact health is by acting as a barrier to health care services and other resources [7, 28]. Their disparate impacts point to the need for study and intervention to promote health equity and well-being.

### Racial discrimination among American Indians and Alaska Natives

For AIANs, contemporary racial discrimination is an extension of genocidal history. Today, stereotyping, intergenerational hate, systemic bias, and exposure to environmental microaggressions (e.g., seeing images in the news or elsewhere that are reminders of racist ideology, such as the Washington Football Team’s former racist name and logo) are pervasive. Yet there are also continual grassroots movements to address racism and stigma. Indigenous scholars posit that historical and contemporary discrimination contribute to health inequities for AI/ANs ([12]; Walters and Simoni, 2002 [58]). Similar to research with other populations, discrimination within health care institutions and from health care providers acts as a barrier to health care and contributes to avoidance of seeking health care services [13, 35, 61]. Moreover, discrimination contributes to substance misuse, including early onset use [59, 60], diabetes-related distress [51], diminished self-care [16], pain, and physical impairment [5]. Further, microaggression exposure within school settings appears to contribute to perception of invisibility and lack of safety [27].

### Assessment of racial discrimination and microaggression exposure

Exposure to discrimination and microaggressions are most commonly assessed in research via self-report measures and with African American samples [1], with relatively few measures that consider the unique racial/ethnic group experiences for AI/ANs (e.g., being told “you don’t look Indian”). Although some recent studies describe racial discrimination measures among AI/ANs (e.g., [17, 49, 55]), there has been limited replication or psychometric evaluation (see [17], for an exception).

We address this critique by using psychometric analyses, including Item Response Theory (IRT) and confirmatory factor analysis (CFA), to evaluate the psychometric properties of three racial discrimination/microaggressions measures among AI/AN college students in a large Southwestern city. Two of the measures were designed for use with any racial/ethnic group (Everyday Discrimination Scale [63]; Experiences of Discrimination [32];) and one measure is an AI/AN-specific discrimination and microaggressions scale (Microaggressions Distress Scale, [57]). While the Everyday Discrimination Scale is the most common measure of discrimination across racial and ethnic groups, only four studies with Indigenous samples have been conducted [17, 49, 50, 55], sometimes with varying items or instructions.

### Study aims

Racial discrimination, including microaggressions, contributes to health inequities, yet research has focused on single measures without adequate psychometric evaluation or inquiry into the severity of the forms of discrimination they measure. To address this gap, we conducted psychometric analyses of three measures of racial discrimination and microaggressions among AI/AN college students in the Southwest United States, bringing these three measures together for the first time. Measurement research is one avenue among many to address racial injustice. The goals of this study included (1) testing the dimensionality of the three measures, (2) characterizing their item- and scale-level properties via IRT and CFA, and (3) examining correlations among scale scores of the three measures and their associations with sociodemographic factors (age, gender, income), substance use, and physical health.

## Method

### Participants

Participants (*N* = 347) were AI/AN students attending public higher education institutions (one university, one community college) in a large southwestern U.S. urban area. The population of the state where the schools are located is 14% AI/AN (U.S. Census, 2010). At the time of the survey, the schools themselves had an AI/AN student population of approximately 6%. Participants’ ages ranged from 18 to 65 years; average age was in the late 20s (Table 1). Approximately two-thirds were women. All participants were AI/AN and 90% were from Southwest tribes. About two-thirds attended the community college; the remaining one-third attended the university. The median annual household income was between $10,000 and $29,999.

**Table 1:** Descriptive Statistics

Measure	M	(SD)
Experiences of Discrimination (total)	1.83	(2.19)
Microaggressions Distress Scale (total)	3.68	(2.25)
Revised-Everyday Discrimination Scale (total)	8.96	(6.34)
Age (years)	28.45	(9.97)
Self-reported physical health (0–4)	2.66	(0.91)
	N	(%)
Female	227	(65.6%)
CAGE-AID (positive)	129	(37.8%)
Current tobacco (everyday or some days)	44	(12.7%)

Eligibility criteria included: (a) enrolled at least part-time in school, (b) 18 years or older, (c) one-quarter AI/AN or tribally enrolled, and (d) physically in the Southwestern city while completing the survey. Greenfield et al. [19] provides further study details.

### Procedure

Ethics approval for this study was provided by the university (12–267) and the community college (Greenfield011113; names of institutions not included to protect confidentiality). A community advisory board of AI/AN faculty, students, and staff from the two institutions provided regular guidance on study design, data collection and interpretation, and dissemination of results, including this article. These advisory board members were selected because they had direct lived experience navigating the two institutions from which participants were recruited.

Survey data were collected between February and July of 2013. The sampling frame included all students listed as American Indian and Alaska Native at the community college and university according to academic records provided by the administration. All AI/AN students enrolled at the community college and the university were sent an email inviting them to participate. Participants also were recruited via announcements at AI/AN student organization meetings on campus, flyers, and word-of-mouth from community advisory board members. Students completed a one-time online survey with measures of discrimination, substance use, and sociodemographic factors. Before accessing the online survey, individuals were shown a consent form to read and review. The document informed them that by clicking the button at the bottom to continue to the next page, they were giving consent to participate. Upon survey completion, participants could enter a gift card raffle, with gift cards totaling $2035 and ranging in value from $5 to $200. Eighty-three participants (23.9%) received a gift card for their study participation.

### Measures

#### Demographics

Participants provided their age, gender, and annual household income (Table 1). Annual household income options were (0) less than $5000, (1) $5000-9999, (2) $10,000-29,999, (3) $30,000-49,999, or (4) $50,000 and above.

#### Revised-Everyday Discrimination Scale

The commonly used Everyday Discrimination Scale was developed with African Americans. It employs a two-stage question stem approach by first asking about the frequency of different types of discrimination (e.g., how often “people act like you are not as smart”), and then about *why* the person thought these experiences happened (e.g., gender, height, race, etc.). Kim et al. [30] found measurement invariance across Hispanic/Latino, Asian, non-Hispanic White, and African American individuals, except for one item (how often “people act as if they are better than you”). Gonzalez and colleagues (2016) later changed the measure from a two- to one-stage approach by altering the question stem to attribute the experience to being AI/AN, but retained all nine items. Everyday Discrimination Scale scores were associated with psychological distress and anger [17]. They did not complete factor analyses of the measure. Additionally, Everyday Discrimination Scale scores have been associated with chronic health concerns and behavioral risk factors for Indigenous samples [49, 55].

Stucky et al. [53] completed an IRT analysis of the original Everyday Discrimination Scale with an African American sample and put forth a five-item revised version of the measure, the r-Everyday Discrimination Scale, which was used here. The r-Everyday Discrimination Scale has demonstrated good convergent validity, good predictive validity, and adequate internal consistency (α = 0.82 to 0.84 [53];). The r-Everyday Discrimination Scale retains the two-stage question approach. As in the original Everyday Discrimination Scale, items are answered on a six-point scale of (0) never, (1) less than once a year, (2) a few times a year, (3) a few times a month, (4) at least once a week, and (5) almost every day. Responses to these five items were summed for a possible score from 0 to 25, with higher scores indicating more experiences of discrimination (Cronbach’s α in this sample = 0.90). At the end of the measure, participants indicated the reason(s) why they thought these experiences happened, out of 14 possible reasons (e.g., ancestry, gender, race).

#### Microaggressions Distress Scale

The Microaggressions Distress Scale [57] included 10 questions about past-year subtle and overt microaggressions (e.g., “told by non-Natives that they felt a spiritual connection to Indians”; “hit or physically attacked because you are Native”). It also included a follow-up question about distress related to each microaggression. The Microaggressions Distress Scale was developed to measure AI/AN-specific microaggressions. An earlier version focused on distress versus frequency ratings and demonstrated good internal reliability (α = 0.97). Higher distress scores were associated with higher odds of physical pain and impairment [5]. Here, response options included (0) “no,” (1) “I’m not sure but I think so,” and (2) “yes.” Options (1) and (2) were combined to create dichotomous response categories (0) “no” and (1) “yes” or “not sure but think so,” because microaggressions often involve some degree of uncertainty.^1^ An answer of “not sure but think so” matches typical microaggression experiences. Responses were summed for a total score from 0 to 10, representing the number of different past-year microaggressions (higher scores indicating more experiences of microaggressions, α in this sample = 0.67).

#### Experiences of Discrimination Measure

The Experiences of Discrimination measure is a nine-item self-report measure about lifetime experiences of racial discrimination attributed to race, ethnicity, or skin color (e.g., school, loan, medical care). For affirmative responses, respondents indicate lifetime frequency as once, 2 to 3 times, or 4 or more times. Krieger et al. [32] developed the Experiences of Discrimination Measure for the Coronary Artery Risk Development in Young Adults (CARDIA) study, which included Black, Latino, and White participants [32]. The measure demonstrated good internal reliability and test-retest reliability. It also correlated with psychological distress and cigarette smoking [32]. In line with Krieger et al. [32], we utilized binary indicators of any experiences with discrimination across the situations reflected by each item (versus no experiences with discrimination for each item) as the primary focus of our analysis, which can be summed to represent the number of different lifetime experiences of racial discrimination (higher scores indicating more experiences of discrimination, α in this sample = 0.79).

#### Tobacco use

A single question adapted from the Behavioral Risk Factor Surveillance System measured current tobacco use [4]. Response options included “everyday,” “some days,” “former smoker,” “not former smoker but have smoked in the past,” “never smoked,” and “only smoke for ceremonial purposes.” Then a single binary variable was created from these responses to indicate current tobacco use (i.e., tobacco use “everyday” or “some days”) for non-ceremonial purposes.

#### CAGE-AID

The CAGE-AID (acronym for cut down, annoyed, guilty, eye opener; adapted to include drugs) is a four-item alcohol or drug use disorder screening measure [6]. For example, one item is, “have you ever felt that you ought to cut down on your drinking or drug use?” Scores of two or more indicate potentially problematic substance use (range 0–4).

#### Self-reported physical health

Response options to the perceived current health item included (0) poor, (1) fair, (2) good, (3) very good, and (4) excellent.

### Data analytic plan

Psychometric properties for the three racial discrimination measures were assessed in a three-step process by first examining scale descriptive statistics and unidimensionality, followed by item-level measurement properties tested using IRT or CFA, then scale-level associations with other constructs. Item-level properties were tested using IRT for the Microaggressions Distress Scale and the Experiences of Discrimination scale, which have binary response options, and using CFA for the revised-Everyday Discrimination Scale, which has Likert-type response options. IRT and CFA are both latent trait models^2^ that evaluate how well individual items within each questionnaire assessed an underlying latent value representing the severity of racial discrimination. As opposed to measuring scale-level reliability only (e.g., using classical test theory methods), the use of IRT and CFA allowed us to understand both full-scale and item-level properties, such as the probability of specific individuals endorsing a questionnaire item in IRT models (i.e., the probability of endorsing specific experiences of racial discrimination as described in each questionnaire item) based on the overall latent level of racial discrimination they have experienced and the degree to which specific items loaded onto a latent variable reflecting discrimination.

IRT analyses used a two-parameter logistic model where the probabilities of endorsing specific items within a measure are expressed through parameters reflecting the latent variable (*θ,* which has a mean of 0 and standard deviation of 1), and two item-level characteristics, including an item *severity* parameter (*b* statistic), which describes the severity of the latent variable where a particular item is most informative (e.g., items with higher severity values may indicate more severe forms of racial discrimination, and items with lower severity values may indicate less severe forms of racial discrimination) and an item discrimination parameter (*a* statistic), which indicates how well an item differentiates individuals with higher versus lower levels of the latent variable near the item’s severity (e.g., items with higher discrimination values are more strongly correlated with the latent racial discrimination variable being measured, and items with lower discrimination values are less strongly correlated with the latent racial discrimination variable). These analyses assume the items within the measure represent a unidimensional characteristic (i.e., as opposed to multiple dimensions), are locally independent (i.e., that correlations among item residuals are small after controlling for the latent characteristic), and monotonic (i.e., that the probability of endorsing them increases as the latent trait increases). Together, the severity and discrimination terms can be used to derive *item characteristic curves*, which graphically illustrate the probability (*p*) of endorsing questionnaire items (*x*) along all levels of the latent trait *θ* using the following formula, where *i* subscripts indicate items within the test and *j* subscripts indicate participants within the sample:

$$ {p}_{ij}\left({\theta}_j\right)=\frac{\mathit{\exp}\left({a}_i\left({\theta}_j-{b}_i\right)\right)}{1+\mathit{\exp}\left({a}_i\left({\theta}_j-{b}_i\right)\right)} $$

These parameter estimates also can be used to derive *item information curves*, which illustrate the degree to which items provide the greatest ability to reliably differentiate between individuals along levels of the latent trait *θ*. The two-parameter logistic IRT models were fit using the *ltm* package [46] in R [44]. Unidimensionality tests were performed using parallel analysis methods that compared the second eigenvalue of the tetrachoric correlation matrix of dichotomous items to the distribution of second eigenvalues simulated under the assumed unidimensional IRT model [8, 46].

CFA with a single latent trait loading onto the five items in the r-Everyday Discrimination Scale was fit using the lavaan package in R [47]. Unidimensionality of this measure was tested using McDonald’s *ω*_*h*_ [66], which assessed the proportion of variance in scale scores accounted for by a general factor within the context of a second-order factor analysis. All models were fit using marginal maximum likelihood, which includes participants with missing data for some response items. Less than 1% of responses had missing data.

Following item-level analyses, we evaluated scale-level properties from the summed scale scores of each measure. Although summed scores are imperfectly correlated with scaled scores derived by IRT and CFA, we chose to evaluate summed scores as we anticipate summed scores would be more accessible and more commonly used in future applied discrimination research and in real-world practice where the computation of latent variables may not be possible. Cross-sectional associations among the summed scale scores and their associations with other measures (age, gender, income, self-rated health, CAGE-AID, tobacco use) were assessed using bivariate correlations. *P*-values in the resulting correlation matrix were adjusted for multiple comparisons using the serial adjustment procedure described by Holm [21] via the R *psych* package [45].

## Results

### Descriptive statistics

#### Substance use and health status

Substance use and health status measures are presented in Table 1. Thirteen percent of participants reported current tobacco use (i.e., every day or some days). Thirty-eight percent had scores of two or higher on the CAGE-AID, indicating a probable lifetime history of problematic substance use. Ninety percent reported current good, very good, or excellent physical health.

#### Racial discrimination and microaggressions

On the Microaggressions Distress Scale, participants experienced an average of 3.68 of 10 possible distinct microaggressions in the past year (*SD* = 2.25; range 0–10; Table 1). They reported an average of 1.83 of nine lifetime racial discrimination experiences (SD = 2.19; range = 0–9) on the Experiences of Discrimination measure. The average score on the r-Everyday Discrimination Scale was 8.96 out of a possible 25 (*SD* = 6.34; range = 0–25). The most common attributions for r-Everyday Discrimination Scale items were race (45%), skin color (38%), gender (32%), age (32%), income (31%), education (29%), and “some other aspect of your physical appearance” (28%).

### Dimensionality testing and latent trait analyses

#### Dimensionality

Dimensionality tests indicated that each measure assessed a single latent variable. The second eigenvalues for the observed data did not differ significantly from the second eigenvalues of simulated unidimensional data for the Experiences of Discrimination measure (observed eigenvalue = 0.53, simulated = 0.59, *p* = .74) and the Microaggressions Distress Scale (observed = 1.13, simulated = 1.13, *p* = .54). For the r-Everyday Discrimination Scale, McDonald’s *ω*_*h*_ = .86, indicating 86% of variance in the summed scale scores was accounted for by a single unidimensional factor underlying all items. We conducted additional unidimensionality tests by combining the three questionnaires.^3^ The unidimensionality of these combined measures was no longer retained when any two scales were combined into a single scale (all *p* < .059). These scales measure overlapping yet distinct dimensions of discrimination and were modeled in separate latent trait models to retain the assumptions of unidimensionality in IRT.

Model fit indices for each unidimensional latent trait model are shown in Table 2. Root mean square error of approximation indicated good fit (< 0.10) for the Microaggressions Distress Scale and Experiences of Discrimination Scale, but less-than-good fit for the revised-Everyday Discrimination Scale (= 0.13). Standardized root measure square residuals indicated good fit (< 0.08, Hu & Bentler, 1999 [23]) for all three measures. Comparative fit indices indicated good fit (> 0.90, [31]) for the revised-Everyday Discrimination Scale and Experiences of Discrimination Scale, but less-than-good fit for the Microaggressions Distress Scale (= 0.88). For all three scales, two of the three fit indices indicated good fit. Further modifications were not made because (1) research has indicated that model-misspecification can result when modifications are made due to rigidly interpreting model fit cutoffs [42], (2) we aimed to retain the measures that were utilized provided model fit was generally adequate, (3) modifying the model (e.g., eliminating items, adding correlated residuals) increases the risk error due to exploratory modifications [25], and (4) deviations from “good” fit in this case were modest.

**Table 2:** Model fit indices for unidimensional latent trait models

	RMSEA (90% CI)	SRMR	CFI	Reliability	Range of residual correlations
Revised-Everyday Discrimination Scale	0.13 (0.09, 0.17)	0.03	0.97	0.90	(−0.09, 0.12)
Microaggressions Distress Scale	0.07 (0.06, 0.09)	0.07	0.88	0.69	(−0.13, 0.15)
Experiences of Discrimination Scale	0.04 (0.01, 0.06)	0.05	0.98	0.67	(−0.13, 0.07)

*Note*. *RMSEA* Root mean square error of approximation, *SRMR* Standardized root mean square residual, *CFI* Comparative fit index, *Reliability* IRT or CFA model-based reliability estimate

Residual correlations between items were low (< 0.15 in magnitude) and all discrimination parameters and factor loadings were positive and significant, as described more below. IRT and CFA model-based reliability estimates were high for the revised-Everyday Discrimination Scale (0.90) and moderate for the Microaggressions Distress Scale (0.69) and Experiences of Discrimination Scale (0.67). Bivariate correlations between items are available in Supplementary file 1.

#### Experiences of discrimination measure

**Fig. 1:**
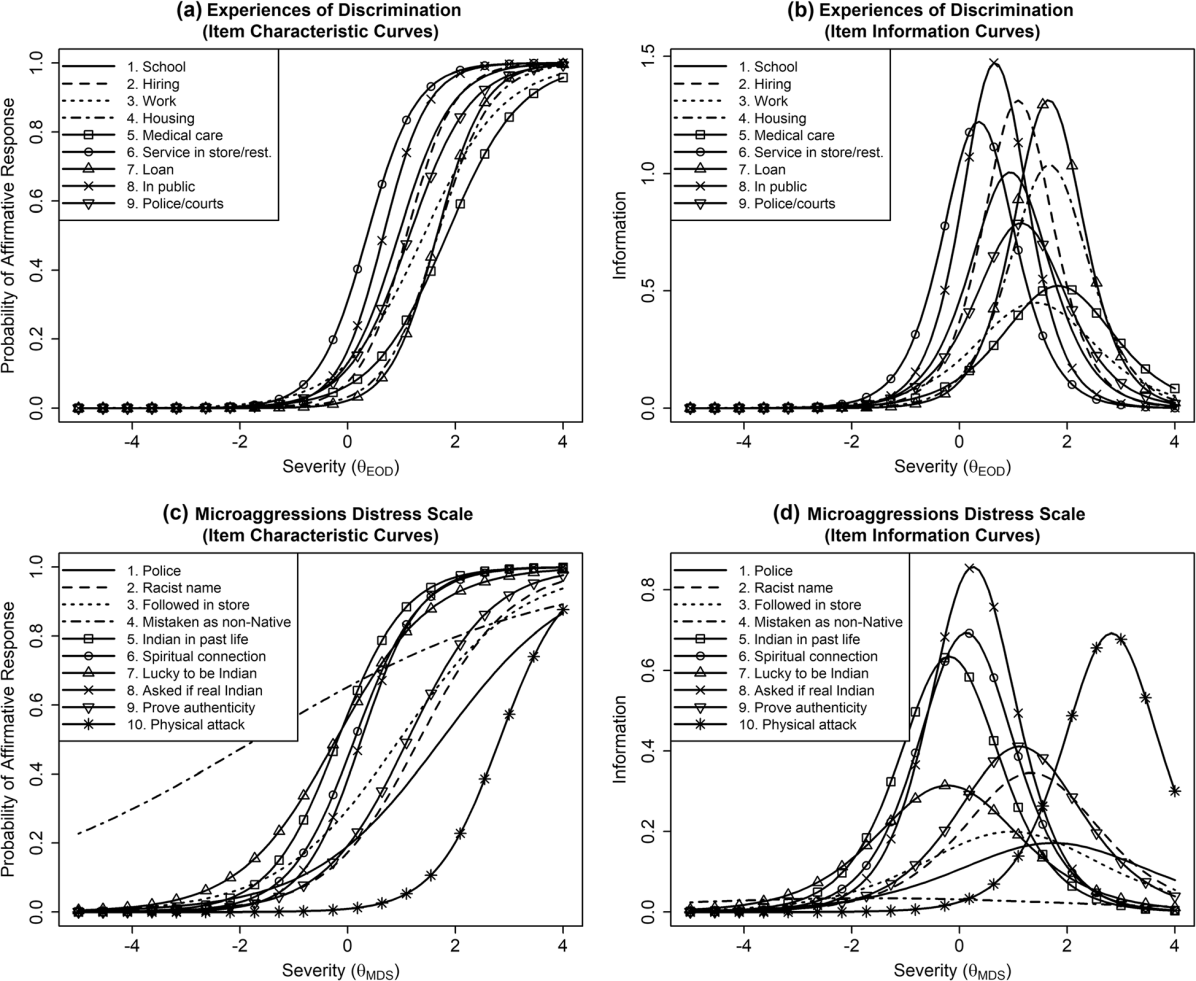
Item characteristic curves (left panels) and item information curves (right panels) for the EOD measure (top), MDS (middle), and r-EDS (bottom). Item characteristic curves for the r-EDS characterize responses indicating any experience with the measured items (i.e., differentiating “never” from all other responses); item information curves for this measure reflect item information across all of the ordinal response options for each item. Different subscripts for θ are used to indicate the different latent variables represented by each measure

Item-level descriptive statistics and IRT parameter estimates are presented in Table 3. Item characteristic curves and item information curves for each measure are presented in Fig. 1. For the Experiences of Discrimination measure, less than 40 % of participants endorsed the racial discrimination noted in any individual item (range = 9.3 to 39.0% across items). IRT severity parameter estimates (*b* column in Table 3) for the Experiences of Discrimination items were all high (range = 0.36 to 1.84), indicating that the Experiences of Discrimination items assessed relatively more severe levels of racial discrimination. Discrimination parameter estimates (*a* column in Table 3) for the Experiences of Discrimination items were also high (range = 1.34 to 2.43), indicating that items were good at differentiating higher versus lower levels of latent discrimination, specifically for those higher on the latent discrimination variable (i.e., participants at higher levels of *θ*).

**Table 3:** Item Level Characteristics for IRT Models (Microaggressions Distress Scale & Experiences of Discrimination scale)

Item	Endorsement	Discrimination		Severity	
	%	*a*	(SE)	*b*	(SE)
Experiences of Discrimination Scale					
1. School	23.6%	2.01	(0.34)	0.94	(0.12)
2. Hiring	19.1%	2.29	(0.41)	1.09	(0.13)
3. Work	19.1%	1.34	(0.25)	1.41	(0.21)
4. Housing	10.2%	2.04	(0.40)	1.67	(0.20)
5. Medical care	12.0%	1.44	(0.28)	1.84	(0.26)
6. Store/restaurant service	39.0%	2.21	(0.37)	0.36	(0.09)
7. Loan	9.3%	2.29	(0.46)	1.65	(0.18)
8. In public	29.5%	2.43	(0.43)	0.66	(0.10)
9. Police/courts	20.4%	1.78	(0.31)	1.15	(0.15)
Microaggressions Distress Scale					
1. Police	21.9%	0.83	(0.20)	1.74	(0.37)
2. Racist name	22.3%	1.18	(0.24)	1.33	(0.22)
3. Followed in store	32.4%	0.89	(0.19)	0.96	(0.21)
4. Mistaken as non-Native	64.7%	0.37	(0.15)	−1.70	(0.71)
5. Indian in past life/Cherokee princess	55.1%	1.59	(0.28)	−0.18	(0.10)
6. Spiritual connection	46.5%	1.67	(0.30)	0.13	(0.10)
7. Lucky to be Indian	54.9%	1.12	(0.20)	−0.22	(0.12)
8. Asked if real Indian	42.7%	1.85	(0.35)	0.25	(0.09)
9. Prove authenticity	24.9%	1.28	(0.25)	1.12	(0.18)
10. Physical attack	2.9%	1.66	(0.50)	2.82	(0.53)

**Fig. 2:**
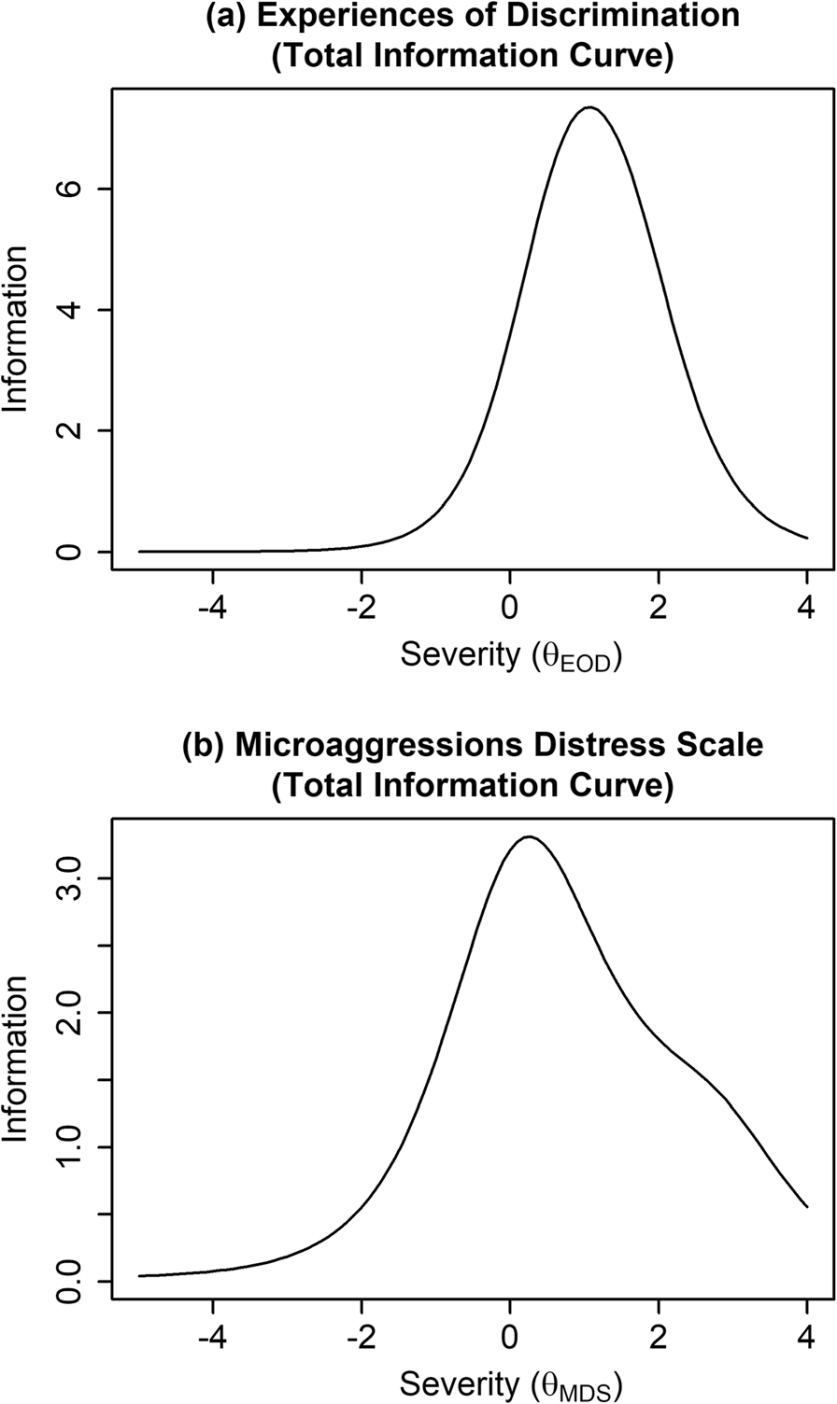
Total information curves for the EOD measure (top), MDS (middle), and r-EDS (bottom). Different subscripts for θ are used to indicate the different latent variables represented by each measure

Item characteristic curves and item information curves for the Experiences of Discrimination measure are displayed in the top-left and top-right panels of Fig. 1, respectively. As shown by the item characteristic curves (top-left panel), participants with average levels of the latent racial discrimination severity variable (*x*-axis) had a low probability of endorsing each of the Experiences of Discrimination items. Participants had only a 50% or more probability of endorsing items when they were 0.36 to 1.84 standard deviations above the mean level of the latent discrimination severity variable. As shown by the item information curves (top-right panel), Experiences of Discrimination items provided the greatest information (i.e., ability to reliably differentiate participants who fall within a specific range of racial discrimination severity) when participants had above-average levels of the latent racial discrimination severity variable (*θ*). In other words, the Experiences of Discrimination provided the most reliable information about a person’s experiences of discrimination (relative to other individuals) when they reported a relatively high level of discrimination. Likewise, the measure provided less reliable information about a person’s experiences with discrimination (relative to other individuals) when they reported relatively lower levels of discrimination. The amount of information varied by item, with items 3 (at work) and 5 (getting medical care) providing the least information. Items 8 (in public), 2 (hiring), 7 (loan), and 6 (service in store/restaurant) provided the most information (i.e., greater ability to differentiate severity of racial discrimination). The amount of total information provided across all items is shown in the top panel of Fig. 2.

#### Microaggressions distress scale

For the Microaggressions Distress Scale, the proportion of participants endorsing each item varied considerably more (range = 2.9 to 64.7%). IRT severity parameter estimates (*b* column in Table 3) were likewise more variable (range = − 1.70 to 2.82), indicating the Microaggressions Distress Scale contained items that captured a relatively wide range of severity of microaggression experiences. Discrimination parameter estimates (*a* column in Table 3) were also more variable (range = 0.37 to 1.85), indicating that some items were less able to differentiate individuals who were higher versus lower on the latent microaggressions variable (*θ*). As shown in item information curves (middle-right panel of Fig. 1), being mistaken as non-Native (item 4) provided almost no information about an individual’s relative severity of microaggressions. Unfair treatment by police (item 1) and being followed in a store (item 3) also provided relatively low information. In contrast, being asked if one was a “real Indian” (item 8), being told the speaker had a spiritual connection to Native people (item 6), being told the speaker was an Indian in a past life (item 5), and being told they were lucky to be Indian (item 7) provided more information (i.e., greater ability to reliably differentiate one’s relative severity of microaggressions) among participants who experienced about an average severity of microaggressions (i.e., *θ* close to 0). Being asked to prove one’s authenticity (item 9) and being called a racist name (item 2) provided more information for participants who experienced above-average severity of microaggressions, and being physically attacked provided the most information for individuals with a very high severity of microaggressions. The amount of total information provided across all items is shown in the bottom panel of Fig. 2.

#### r-Everyday discrimination scale

CFA results for the r-Everyday Discrimination Scale are also presented in Table 4. Most participants experienced the form of everyday discrimination noted in each item of the r-Everyday Discrimination Scale at least “less than once a year” or more (i.e., responses greater than “never” ranged from 62.0 to 86.9%). Factor loadings ranged from 0.71 to 0.88, indicating that all items loaded onto the latent factor positively and to a somewhat similar degree, albeit with a slightly lower loading for the names/insulted item compared to the rest. Item intercepts ranged from 0.94 to 1.60, which reflect the expected values for each item (on a 0 to 5 scale) for a person with a latent variable value of 0 (i.e., mean level of everyday discrimination within the sample). Residual variances were modest (0.31 to 0.49) and in line with results that are commonly encountered in CFA.

**Table 4:** Factor Loadings for CFA Model (revised-Everyday Discrimination Scale)

	Loading	(SE)	Intercept	(SE)	Residual Variance	(SE)	Percent of sample reporting this form of discrimination in past year
1. Not smart	0.80	(0.02)	1.18	(0.07)	0.37	(0.04)	71.0%
2. Better than you	0.88	(0.02)	1.60	(0.08)	0.31	(0.04)	86.9%
3. Dishonest	0.81	(0.02)	1.04	(0.07)	0.35	(0.04)	67.4%
4. Less respect	0.83	(0.02)	1.17	(0.07)	0.32	(0.04)	72.4%
5. Names/insulted	0.71	(0.03)	0.94	(0.07)	0.49	(0.04)	62.0%

*Note*. Parameter estimates reflect those obtained from the standardized solution of the CFA model

### Associations with other measures

Table 5 presents correlations among the discrimination measures and other measures. The three discrimination measures had moderate to large correlations with one another (range = 0.41 to 0.62, all *p* < .001), supporting the hypothesis that they tap into a similar overarching construct (i.e., discrimination) while still capturing different aspects of discrimination experiences. In general, more experiences of discrimination were associated with poorer physical health and lower household income. Gender was unrelated to discrimination. CAGE-AID scores were positively correlated with all three measures. Self-reported physical health was negatively associated with the Experiences of Discrimination Scale and the r-Everyday Discrimination Scale, but unrelated to the Microaggressions Distress Scale.

**Table 5:** Correlations of Discrimination, Demographics, and Substance Use

	1.	2.	3.	4.	5.	6.	7.	8.
1. EOD total								
2. MDS total	.62***							
3. r-EDS total	.41***	.41***						
4. Age	.14**	−.13*	−.08					
6. Gender (female)	−.04	−.02	.01	−.01				
5. Income	−.22***	−.14*	−.19***	.15**	−.02			
6. Health	−.17**	−.03	−.13*	−.01	.00	.13*		
7. CAGE-AID	.23***	.15**	.16**	.22***	−.21***	−.12*	−.11*	
8. Current Tobacco	.26***	.15**	**.10**	.04	−.18***	−.16**	−.14*	.17**

*Note*. Measures significantly correlated with discrimination measures are in bold font. *EOD* Experiences of Discrimination, *MDS* Microaggressions Distress Scale, *r-EDS* Revised-Everyday Discrimination Scale, *CAGE-AID* Positive score on the CAGE-AID, indicating problematic lifetime substance use. *p < .05, ** *p* < .01, *** *p* < .001

## Discussion

This study is the first to assess the dimensionality of behavioral manifestations of racial attitudes and stereotypes that impact AI/ANs using three measures of discrimination: the Experiences of Discrimination Scale, the r-Everyday Discrimination Scale, and the Microaggressions Distress Scale. We tested dimensionality (Aim 1), item- and scale-level properties across the measures (Aim 2), and associations with health-related correlates within AI/AN college students in the southwest U.S. (Aim 3). Unidimensionality tests indicated that the three measures tapped into overlapping, yet semi-distinct constructs (i.e., moderately correlated). Existing measures may tap into various forms of discrimination along a continuum of severity. Latent variable models also indicated the individual discrimination experiences underlying items within these measures existed along continua of severity (Aim 2). A greater severity of discrimination was associated with problematic substance use and poorer self-reported physical health (Aim 3).

### Research and theoretical implications

Our findings inform the measurement of racial discrimination, including microaggressions. Using latent variable models, we found that different forms of discrimination exist on continua of severity. Most individuals were likely to experience discrimination described by the r-Everyday Discrimination Scale and the Microaggressions Distress Scale; however, overt forms of discrimination from the Experiences of Discrimination measure (e.g., prevented from doing something or hassled at school, work, or while getting housing) were somewhat less frequently reported. Endorsement of the Microaggressions Distress Scale items may vary based on local or educational context (e.g., Hispanic serving institution, tribal college). Findings from the current study can inform assessment of discrimination in research with AI/ANs, particularly for AI/ANs in higher educational settings. For example, discrimination experiences can be measured on a continuum from common/less severe (e.g., microaggressions and everyday discrimination) to less frequent/more severe (e.g., discrimination impacting school, work, or housing) in line with the severity parameters identified here. In unstructured assessment settings (e.g., unstructured interviews with AI/AN individuals who report discrimination), one may take an adaptive approach by first asking about intermediately severe experience, then based on the response, ask about more or less severe experiences, including the range of experiences represented in the three measures tested here. Because these are trauma-related experiences, making sure the individual is willing to talk about them and feels safe doing so is imperative. At the same time, there still may be variability in how individuals are impacted by microaggressions and discrimination, thus during assessments, it is worth inquiring about how individuals experience them (asking about impact rather than assuming a certain level of severity). From a clinical perspective, assessment questions can tap into a variety of forms and levels of severity of discrimination experiences to more fully understand the client experience.

Our work suggests a need for better conceptualization of microaggressions. IRT analyses identified some Microaggressions Distress Scale items with distinct item-level properties that could be used in future measure refinement. For example, the item assessing whether participants were mistaken as non-Native provided almost no information about the underlying level of microaggressions a person experienced (i.e., low IRT discrimination parameter). For AI/ANs in the southwest U.S., being mistaken as non-Native may be a common experience minimally associated with other types of microaggressions. Unfair police treatment and being followed in a store had somewhat higher IRT discrimination parameters and the overall amount of information about microaggression experiences was lower than other items.^4^ Finally, while the Microaggressions Distress Scale item assessing physical attacks had a high IRT discrimination parameter estimate, it nonetheless reflects a very severe form of discrimination that only participants who experienced very severe levels of microaggressions (i.e., high scores on this measure) would likely endorse.

The relationship between past-year microaggressions, lifetime drug use, and problematic substance use (CAGE-AID score) suggest discrimination is related to substance use. This adds to the growing literature linking discrimination to poorer health [64]. In addition, participants with lower household incomes reported more past-year discrimination and microaggressions which is similar to research findings regarding the lived experiences of African Americans [62]. Similar to prior AI/AN research (e.g., [29]), there were no gender differences in frequency of racial discrimination.

### Clinical and policy implications

Our work supports previous findings that racial discrimination, including exposure to miscroagressions, is pervasive and negatively impacts health. Nearly the entire sample reported exposure to at least one microaggression or other instance of discrimination in the past year. Similar rates have been reported in previous studies with AI/AN young adults and African Americans [29, 33]. Microaggressions have been associated with lower self-esteem in college students [37] as well as poorer health and greater substance use in population-based studies [64]. Future work should further specify the relationship between discrimination, substance use, and other health outcomes, in part through improved measurement.

Identifying and implementing interventions to address discrimination at individual, community, population, and institutional levels is an important goal for improving AI/AN health specifically, and health of BIPOC, generally. Creative, multi-level strategies are needed to reduce exposure to discrimination and microaggressions. Strategies could include addressing implicit and explicit bias such as shifting the underlying attitudes that undergird these forms of bias. Implicit bias generally functions as an unconscious process and fosters negative group associations and attitudes [9]; thus, this form of bias is more likely to be aligned with and allow space for microaggression exposures to proliferate. Tribal communities and institutions are promoting positive contemporary narratives and images to change perceptions and attitudes [10]. Explicit bias reflects conscious mental processes, generally resulting in deliberate harmful acts of discrimination [9]. Both forms of bias lead to discrimination that have harmful effects [9, 43].

Because attitude and policy shifts may occur at a slow pace, we touch upon ways to support marginalized and oppressed communities in managing racial discrimination. Practitioners can encourage victims of race-based trauma to seek social support [3] and encourage allies to speak up when they witness discrimination and microaggressions. This can reduce the burden of effort on BIPOC and has the potential to buffer consequences of experiencing racial discrimination. Interventions to help individuals assess how they respond to individual acts of discrimination and expand their tactics for response may be useful. Also, mindfulness practices could counteract the negative physical effects of discrimination by decreasing cortisol levels [26].

### Limitations and future research

This study focused on discrimination experiences of AI/AN college students in the Southwestern U.S. and found a link between discrimination, substance misuse, and self-reported physical health. Experiences of racial discrimination likely differ for those not in higher education, and by geography, tribal nation, phenotype, age, generation, and ethnic group. Similar to the general literature on discrimination and health (though [15] is an exception), this study was cross-sectional, limiting our ability to determine the causal relationship between discrimination, substance use, and health status. Discrimination also occurs at the institutional level; integrated into systems that BIPOC navigate often. We agree with Williams et al. [64] who have called for the study of multi-level distal effects of systemic racism (e.g., air pollution, food and housing access); beyond interpersonally mediated discrimination. Discrimination likely intersects with these other stressors [12]. Future studies should use longitudinal methods to examine effects of discrimination over the life course and consider measures of implicit bias [43].

Some survey items from the measures in our analyses did not reflect the construct as intended for assessment. For example, one item was related to a physical attack, yet was included in an assessment of microaggression exposures. Finally, we focused only on racial discrimination. Some types of discrimination toward AI/ANs are less about race and more about tribal nation citizenship status. Individuals have multiple intersecting identities which can be targets for biased behaviors (e.g., gender identity, sexual orientation). Inclusion of an expanded assessment of discriminatory experiences would add nuance to our understanding of this topic. It may be that the types of microaggressions faced by AI/ANs for the current generation attending college are different from those of which the measures which was developed (e.g., fewer young adults shop in malls but rather on the internet, more consciousness of Indian mascots). Furthermore, there are other stressors that play a role in the health of AI/ANs. We also did not test whether items functioned differently for specific subgroups due to having a somewhat limited sample size for testing this; future research should evaluate whether these measures perform consistently across pertinent subgroups.

## Conclusions

In clinical practice and in research, the measures evaluated here can index varying levels and types of discrimination for AI/ANs, particularly for those in higher educational settings. Items within the three measures tested here reflected varying severity of experiences with discrimination, from hassles to physical harm, reflecting the tendency for discrimination experiences to exist on a continuum. The measures tested here can be used to quantify discrimination experiences to better understand their potential impacts on health and resilience. Naming and quantifying these experiences is one step towards addressing them, and towards healing.

## Supplementary Information

**Additional file 1.** Contains bivariate correlation tables for the items in each of the three primary measures studied here.

## Data Availability

Datasets analyzed in this study are available by reasonable request to the corresponding author and approval by the study’s community advisory board.

## Abbreviations

AI/ANs: American Indians/Alaska Natives
IRT: Item response theory

## Footnotes

1 Moreover, only 6.7% of items were answered as “not sure but think so”, compared to 30.2% of items answered “yes” and 63.2% of items answered “no”. Although a graded response IRT could be conducted to accommodate all three response options, the resulting parameters would indicate the probabilities of moving from “no” to “not sure but think so” and from “not sure but think so” to “yes”, which we determined was less substantively meaningful than the probabilities of moving from “no” to “yes” and/or “not sure but think so” that would be obtained by combining the latter two responses into a single response.

2 Although experiences of discrimination and microaggressions are not traits of individuals, our utilization of the latent trait model conceptualizes the constructs we measure as latent variables (e.g., overall experiences with discrimination or microaggressions) that are imperfectly measured by a finite set of indicators (i.e., items included in each measure).

3 For this analysis, the ordinal r-Everyday Discrimination Scale responses were converted to binary responses (so all responses would be in a binary scale) by classifying “never” as 0 and all responses greater than “never” as 1.

4 Note that even though these specific microaggressions provided less information for differentiating the severity of overall microaggression experiences a person had, our analyses should not be interpreted as indicating that the consequences of these specific microaggressions are less impactful than other microaggressions.
