# Supplementary material for: Understanding measures of racial discrimination and microaggressions among American Indian and Alaska Native college students in the Southwest United States
Source: BMC Public Health. 2021 Jun 9;21:1099. doi: 10.1186/s12889-021-11036-9 (PMC8190861; doi:10.1186/s12889-021-11036-9)
Supplement: Supplementary file 1 — Additional file 1. Contains bivariate correlation tables for the items in each of the three primary measures studied here. [file 12889_2021_11036_MOESM1_ESM.docx]

**Supplemental Tables:** inter-item correlations

| Inter-item correlations: Experiences of Discrimination Scale | | | | | | | | | |
| --- | --- | --- | --- | --- | --- | --- | --- | --- | --- |
|  |  | 1. | 2. | 3. | 4. | 5. | 6. | 7. | 8. |
| 1. | School |  |  |  |  |  |  |  |  |
| 2. | Hiring | 0.31 |  |  |  |  |  |  |  |
| 3. | Work | 0.29 | 0.36 |  |  |  |  |  |  |
| 4. | Housing | 0.22 | 0.35 | 0.26 |  |  |  |  |  |
| 5. | Medical care | 0.28 | 0.26 | 0.16 | 0.20 |  |  |  |  |
| 6. | Store/restaurant service | 0.36 | 0.37 | 0.18 | 0.32 | 0.18 |  |  |  |
| 7. | Loan | 0.25 | 0.38 | 0.28 | 0.42 | 0.28 | 0.30 |  |  |
| 8. | In public | 0.47 | 0.34 | 0.30 | 0.29 | 0.27 | 0.45 | 0.21 |  |
| 9. | Police/courts | 0.31 | 0.32 | 0.24 | 0.24 | 0.28 | 0.34 | 0.34 | 0.35 |

| Inter-item correlations: Microaggressions Distress Scale | | | | | | | | | | |
| --- | --- | --- | --- | --- | --- | --- | --- | --- | --- | --- |
|  |  | 1. | 2. | 3. | 4. | 5. | 6. | 7. | 8. | 9. |
| 1. | Police |  |  |  |  |  |  |  |  |  |
| 2. | Racist name | 0.22 |  |  |  |  |  |  |  |  |
| 3. | Followed in store | 0.26 | 0.26 |  |  |  |  |  |  |  |
| 4. | Mistaken as non-Native | 0.05 | 0.19 | 0.12 |  |  |  |  |  |  |
| 5. | Indian in past life/Cherokee princess | 0.13 | 0.19 | 0.15 | 0.03 |  |  |  |  |  |
| 6. | Spiritual connection | 0.13 | 0.18 | 0.22 | -0.03 | 0.43 |  |  |  |  |
| 7. | Lucky to be Indian | 0.14 | 0.15 | 0.14 | 0.03 | 0.25 | 0.27 |  |  |  |
| 8. | Asked if real Indian | 0.09 | 0.21 | 0.16 | 0.17 | 0.33 | 0.30 | 0.29 |  |  |
| 9. | Prove authenticity | 0.13 | 0.18 | 0.07 | 0.11 | 0.17 | 0.22 | 0.15 | 0.41 |  |
| 10. | Physical Attack | 0.24 | 0.16 | 0.18 | 0.05 | 0.05 | 0.12 | 0.09 | 0.06 | 0.18 |

| Inter-item correlations: Everyday Discrimination Scale-Revised | | | | | |
| --- | --- | --- | --- | --- | --- |
|  |  | 1. | 2. | 3. | 4. |
| 1. | Not smart |  |  |  |  |
| 2. | Better than you | 0.71 |  |  |  |
| 3. | Dishonest | 0.61 | 0.68 |  |  |
| 4. | Less respect | 0.62 | 0.66 | 0.70 |  |
| 5. | Names/insulted | 0.59 | 0.55 | 0.54 | 0.65 |
